# *ACTN4* and the pathways associated with cell motility and adhesion contribute to the process of lung cancer metastasis to the brain

**DOI:** 10.1186/s12885-015-1295-9

**Published:** 2015-04-12

**Authors:** Yufei Gao, Guanghu Li, Liankun Sun, Yichun He, Xiaoyan Li, Zhi Sun, Jihan Wang, Yang Jiang, Jingwei Shi

**Affiliations:** 1 Department of Neurosurgery, China-Japan Union Hospital, Jilin University, Changchun, 130033 China; 2 Department of Thoracic Surgery, The First Hospital of Jilin University, Changchun, 130021 China; 3 Department of Pathophysiology, College of Basic Medical Sciences, Jilin University, Changchun, 130024 China; 4 School of Stomatology, Jilin University, Changchun, 130021 China; 5 Department of Laboratory Medicine Center, China-Japan Union Hospital, Jilin University, Changchun, 130033 China; 6 Department of Colorectal Surgery, China-Japan Union Hospital, Jilin University, Changchun, 130033 China

**Keywords:** ACTN4, Cytoskeleton organization, Metastasis, Lung cancer, Brain tumor

## Abstract

**Background:**

The aim of this study was to identify critical gene pathways that are associated with lung cancer metastasis to the brain.

**Methods:**

The RNA-Seq approach was used to establish the expression profiles of a primary lung cancer, adjacent benign tissue, and metastatic brain tumor from a single patient. The expression profiles of these three types of tissues were compared to define differentially expressed genes, followed by serial-cluster analysis, gene ontology analysis, pathway analysis, and knowledge-driven network analysis. Reverse transcription–polymerase chain reaction (RT-PCR) was used to validate the expression of essential candidate genes in tissues from ten additional patients.

**Results:**

Differential gene expression among these three types of tissues was classified into multiple clusters according to the patterns of their alterations. Further bioinformatic analysis of these expression profile data showed that the network of the signal transduction pathways related to actin cytoskeleton reorganization, cell migration, and adhesion was associated with lung cancer metastasis to the brain. The expression of *ACTN4* (*actinin, alpha 4*), a cytoskeleton protein gene essential for cytoskeleton organization and cell motility, was significantly elevated in the metastatic brain tumor but not in the primary lung cancer tissue.

**Conclusions:**

The signaling pathways involved in the regulation of cytoskeleton reorganization, cell motility, and focal adhesion play a role in the process of lung cancer metastasis to the brain. The contribution of *ACTN4* to the process of lung cancer metastasis to the brain could be mainly through regulation of actin cytoskeleton reorganization, cell motility, and focal adhesion.

**Electronic supplementary material:**

The online version of this article (doi:10.1186/s12885-015-1295-9) contains supplementary material, which is available to authorized users.

## Background

Metastatic brain tumors are the most common type of brain tumor in adults and are associated with a poor survival of patients (median survival time = 3–6 months) [1]. A total of 40–50% of brain metastases originate from lung cancer [2]. Studies of differential gene expression between brain metastases and primary lung cancer have suggested that many genes may be involved in the brain metastasis of lung cancer. Using a cDNA microarray approach, more than 200 genes, including genes encoding plasma membrane proteins, antigen proteins, and cytoskeletal proteins, have been found to be differentially expressed between a metastatic brain tumor and a lung adenocarcinoma [3]. These genes function in cell interaction, attachment, and motility.

Actinin, alpha 4 (ACTN4), a nonmuscle cytoskeleton protein, has been frequently reported to be associated with cell motility and cancer metastasis. Honda *et al*. have suggested that cytoplasmic ACTN4 increases cell motility and is associated with a high metastatic potential and a poor prognosis of cancer based on their studies on multiple cancer cell lines, including lung cancer cell lines, and 61 patients with early-stage breast cancer [4]. Since then, *ACTN4* has been reported to be associated with the progression and metastasis of many types of cancer, including breast [5], colorectal [6], pancreatic [7], lung [8-10], brain [11], bladder [12,13], and ovarian cancers [14-16] and salivary gland carcinoma [17]. In addition, *ACTN4* was found to be highly expressed in a poor survival group of patients with non-small cell lung cancer, suggesting that *ACTN4* is a significant prognostic predictor in this cohort of patients [8]. A novel alternative splice variant RNA of *ACTN4* has been suggested to be a candidate diagnostic marker of human small cell lung cancer [9] and a prognostic factor for patients with high-grade neuroendocrine pulmonary tumors [10]. However, in contrast to the aforementioned function of *ACTN4* as a positive regulator of tumorigenesis or cancer metastasis, several studies have suggested that *ACTN4* may function like a tumor suppressor to suppress malignant behaviors of cancer cells [18,19]. Therefore, the association of *ACTN4* expression with tumorigenicity and cancer metastasis needs to be further investigated in the clinic.

In order to define the critical signaling pathways and genes that contribute to the brain metastasis of lung cancer, we used the RNA-Seq approach to investigate the expression profiles of three types of tissues (primary lung cancer, adjacent benign lung tissue, and metastatic brain tissue) from one patient. Subsequently, a series of bioinformatic analyses were performed with the RNA-Seq data to identify differentially expressed genes among these three types of tissues and to discover the critical pathways and genes responsible for the brain metastasis of lung cancer.

## Methods

### Study subject

A 47-year-old female patient was found to have a space-occupying lesion in her lung in January 2009. A diagnosis of poorly/moderately differentiated adenocarcinoma with a primary tumor–lymph node–distant metastasis stage of T2N1Mx in the right middle lobe of the lung was made in January 2010. Resection of the lower part of the right middle lobe was conducted and followed by chemotherapy. The metastatic tumor at the right frontal lobe of the brain was found and resected in December 2010. Pathological analyses showed that the tumor was a poorly/moderately differentiated adenocarcinoma that had metastasized from the lung. The adjacent benign lung tissue (N16), the original lung cancer (T16), and the metastatic brain tumor (T30) were collected. This study was approved by the institutional review board of China-Japan Union Hospital of Jilin University and conducted in accordance with the ethical guidelines of the Declaration of Helsinki. The patient had signed a consent form before the study. In addition, we collected lung cancer and para-tumor tissues from ten patients for the confirmation study.

### RNA isolation and RNA-seq library preparation

Total RNA was isolated from the tissues using a Trizol reagent (Invitrogen, Carlsbad, CA, USA). The RNA quality was assessed using a Bioanalyzer 2200 (Agilent, Santa Clara, CA, USA), and the samples were stored at −80°C until use. The RNA integrity numbers (RIN) of these RNA samples were more than 8.0 and were appropriate for cDNA library construction.

### cDNA library construction and sequencing

The TruSeqTM RNA Sample Preparation Kit (Illumina, Inc.) was used to construct the cDNA libraries for these RNA samples, according to the manufacturer’s instructions. Briefly, oligo(dT) magnetic beads were applied to purify mRNA using 10 μg of total RNA, and the purified mRNA was subsequently fragmented into sizes of 200–500 bp using divalent cations at 94°C for 5 min. Reverse transcription (RT) of the first-strand cDNA from the RNA fragments was performed using SuperScript II reverse transcriptase and random primers. The second strand cDNA synthesis was performed using DNA polymerase I and RNase H. The synthesized cDNA fragments were then end-repaired by adding a single “A” base ligated with indexed adapters. These end-repaired cDNA fragments were purified and enriched by the polymerase chain reaction (PCR). The final cDNA libraries were generated by size selection through 2% agarose gel electrophoresis and quantified by a Bioanalyzer 2200. The tagged cDNA libraries were pooled in an equal ratio and loaded in a single lane of the Illumina HiSeqTM 2000 for paired-end sequencing.

### qRT-PCR

The endogenous control *β-actin* was used as a control for RT-PCR amplification measurement of *ACTN4* expression. RT-PCR primers were *β-actin* (5′-CTGGAACGGTGAAGGTGACA-3′ and 5′-AAGGGACTTCCTGTAACAATGCA-3′) and *ACTN4* (5′-ACAAGCCCAACCTGGAC-3′ and 5′-GGTGCGGGCAATGGTG-3′). The cDNA was generated using a High-Capacity cDNA Reverse Transcription kit (Applied Biosystems, Foster City, CA, USA) and oligo(dT) primers, according to the manufacturer’s instructions. qPCR amplification was performed with the following conditions: 2 min at 50°C, 10 min at 95°C, and 50 cycles of 15 s at 95°C, and 1 min at 60°C. The conditions for the melting curve analysis were 1 min at 90°C, 30 s at 55°C, and 30 s at 95°C.

### Bioinformatic analysis

The DEGseq algorithm was applied to filter the differentially expressed genes with a fold change > 2, P < 0.5, and false discovery rate (FDR) < 0.05 [20]. Gene ontology (GO) analysis was performed according to the GO annotations from NCBI (http://www.ncbi.nlm.nih.gov/), UniProt (http://www.uniprot.org/), and Gene Ontology (http://www.geneontology.org/). The pathway analyses were performed to determine the significant pathways associated with the differentially expressed genes according to the KEGG database. Fisher’s exact test, P values, and FDRs were applied in the GO and pathway analyses, according to a previous study [21]. Series cluster analysis was performed to classify the differentially expressed genes in eight clusters based on the reads per kb per million reads (RPKM) change tendency of genes in these three types of tissues (N16, T16, and T30), according to a previous study [22]. For example, the genes with the following expression pattern were classified into Cluster 1: expression in N16 > expression in T16 = expression in T30. The Path-Act-Network analyses were performed to reveal the interactive network among the pathways with enriched differentially expressed genes based on the KEGG database, including the metabolism, membrane transport, signal transduction, and cell cycle pathways [23]. Cytoscape software was used to generate the graphical representations of the pathways [24]. Gene-Act-Net analyses were conducted to reveal the network of the differentially expressed genes based on the interactions among the genes, proteins, and compounds included in the KEGG database.

## Results

### Quality control

Quality control was confirmed using Fast-QC to ensure that the quality scores of the majority of the sequence data were higher than 28 (data not shown), indicating that the data quality was satisfactory for the following analyses. Per sequence GC content curves also showed that the GC distribution from our data matched with the theoretical distribution (data not shown). A total of 32.7 × 10^6^, 43.5 × 10^6^, and 39.1 × 10^6^ reads were obtained for the adjacent benign, primary lung cancer, and metastatic brain tumor tissues, respectively. In addition, the mapping rates were 75.6%, 92.0%, and 91.1% for these three types of tissues, respectively (Table 1).

**Table 1:** **Read numbers and mapping rates for the data from these three types of tissues**

Term	N16	T16	T30
All reads	43279252	47347620	39090277
Mapped reads	32733724	43537602	39090265
Unique mapped reads	30080568	42382073	37298341
Repeat mapped reads	2653166	1155540	1791935
Mapping rates	0.756337563	0.91953095	0.911085382
Unique mapping rates	0.695034378	0.895125732	0.869320616

### Differentially expressed genes among these three types of tissues

The RNA-Seq data from these three types of tissues that had passed the aforementioned quality control were mapped to the reference genome, followed by the statistical analyses and expression analyses based on the RPKM values and upper-quartile normalization (Additional file 1: Table S1), according to a previous study [25]. Subsequently, differentially expressed genes were further analyzed using the DEGSeq method. We found that there were more than 900 differentially expressed genes between N16 and T16 (Additional file 2: Table S2) and more than 800 differentially expressed genes between N16 and T30 (Additional file 3: Table S3). Notably, the expression of *ACTN4* did not show a significant difference between N16 and T16, but it was significantly increased in T30 (P = 2.26 × 10^−17^, FDR = 6.53 × 10^−15^).

### Series-cluster analysis

**Figure 1:**
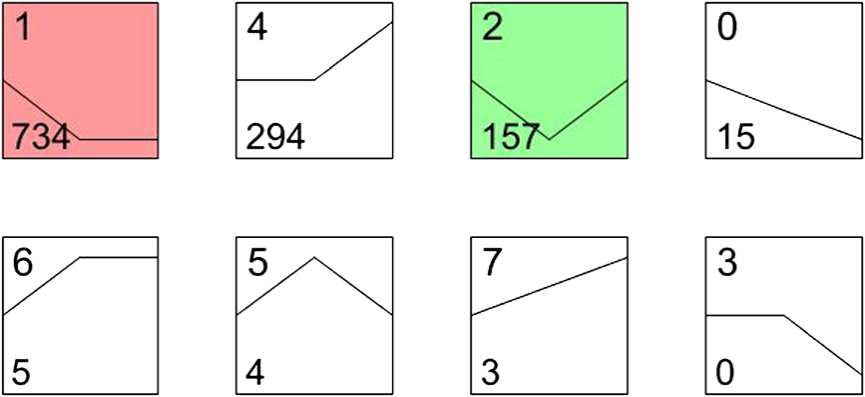
Eight clusters of genes with unique patterns of expression alteration in three types of tissues. Clusters were ordered based on the number of genes assigned. The cluster number is shown at the top left corner of each cluster square. The number of genes grouped in each cluster is shown at the bottom left corner of each cluster square. The distances from the left-end point, the middle point, and the right-end point of the polyline within each cluster square to the bottom line of each square represent the relative (unscaled) gene expression levels among N16, T16, and T30, respectively. For example, the expression levels of genes in cluster 1 were comparable between T16 and T30, but were lower in T16 than in N16.

The series-cluster analysis of these differentially expressed genes classified these genes into eight clusters based on the trend of gene expression among the three types of tissues, i.e., 15 genes in cluster 0 with RPKM N16 > RPKM T16 > RPKM T30, 734 genes in cluster 1 with RPKMN16 > RPKMT16 = RPKMT30, 157 genes in cluster 2 with RPKM N16 > RPKM T16 < RPKM T30, no gene in cluster 3 with RPKMN16 = RPKM T16 > RPKM T30; 294 genes in cluster 4 with RPKM N16 = RPKM T16 < RPKM T30, 4 genes in cluster 5 with RPKMN16 < RPKM T16 > RPKM T30, 5 genes in cluster 6 with RPKM N16 < RPKM T16 = RPKMT30, and 3 genes in cluster 7 with RPKM N16 < RPKM T16 < RPKM T30 (Figure 1). Among them, clusters 1, 4, and 2 were the largest clusters. For example, cluster 1 contained 734 genes, the expression levels of which were significantly reduced in the primary lung cancer tissue compared to that of the benign tissue but was comparable between the primary lung cancer and the brain metastatic tissues. The trend of altered expression among these three types of tissues indicated that these genes possibly play a role in the development of primary lung cancer but may not be critical for brain metastasis. The genes in cluster 4 showed comparable levels of expression in primary lung cancer and the adjacent benign tissues, but they were significantly increased in the metastatic brain tumor. Therefore, these genes are the most likely candidate genes to play an important role in lung cancer metastasis to the brain but may not be critical for lung cancer development. ACTN4 was included in cluster 4 due to its significant increase in the brain tumor tissue but no apparent increase in the lung cancer tissue compared to the adjacent benign tissue.

### Gene Ontology (GO) analysis

**Figure 2:**
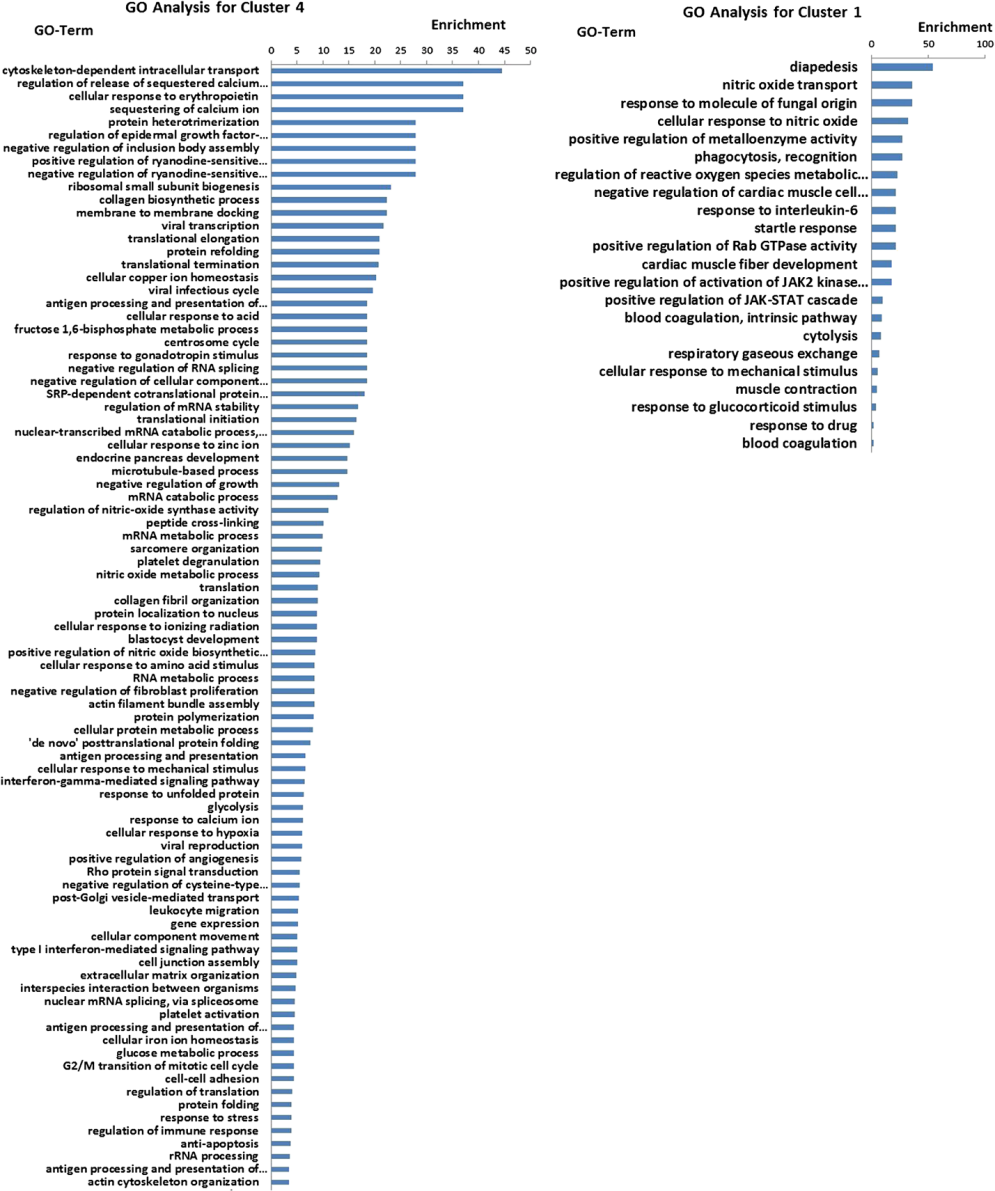
GO analysis for genes in cluster 4 (left) and cluster 1 (right).

In order to explore the gene function relevant to the brain metastasis of lung cancer, GO analysis was conducted to group these differentially expressed genes into signaling pathways. In brief, GO analysis of genes in cluster 4 showed that these genes function in cytoskeleton-dependent intracellular transport, calcium ion transportation, cellular response to erythropoietin, EGFR signal regulation, membrane-to-membrane docking, actin filament bundle assembly, cell-cell adhesion, and actin cytoskeleton organization (Figure 2). Cluster 1 was shown to regulate the reactive oxygen species metabolic process, phagocytosis recognition, response to interlukin-6, positive regulation of Rab GTPase activity, and positive regulation of activation of JAK2 kinase activity and the JAK-STAT cascade (Figure 2).

### Pathway analysis

**Figure 3:**
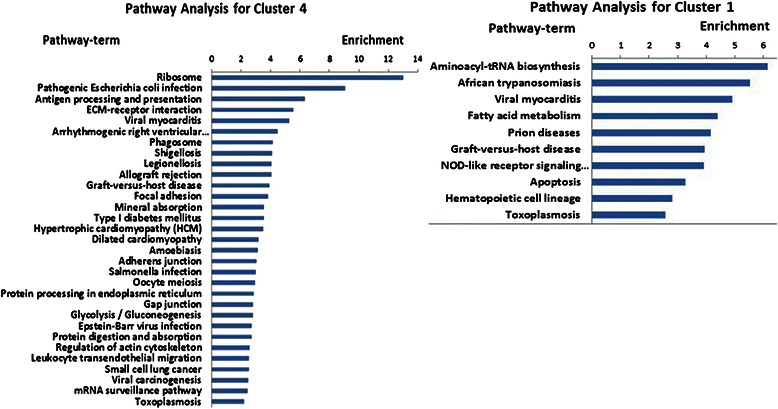
Pathway analysis for genes in cluster 4 (left) and cluster 1 (right).

Further pathway analysis showed that the signaling pathways involved in antigen processing and presentation, extracellular matrix (ECM)-receptor interaction, focal adhesion, adherens junction, glycolysis and gluconeogenesis, regulation of actin cytoskeleton, and small cell lung cancer, etc. are significantly enriched for the genes in cluster 4 (Figure 3). In contrast, the signaling pathways involved in aminoacyl-tRNA biosynthesis, apoptosis, and hematopoietic cell lineage were enriched for the genes in cluster 1 (Figure 3).

### Pathway-Act-Network analysis

**Figure 4:**
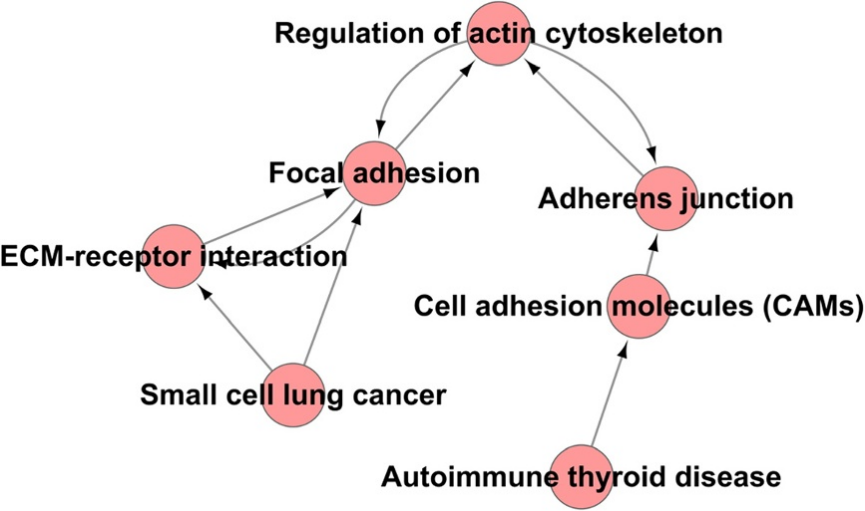
Pathway-Act-Network analysis.

The interactive network among these enriched pathways was subsequently explored. The inferred interactive pathway network indicated that the signaling pathways involved in regulation of actin cytoskeleton, focal adhesion, and adherens junction received stimulation from the signaling pathways related to small cell lung cancer through an ECM-receptor interaction and to autoimmune thyroid disease through cell adhesion molecules (CAMs) (Figure 4). The signaling pathway cascade was revealed by this network. The signaling pathway involved in regulation of actin cytoskeleton appears to be the pivotal point of the Pathway-Act-Network.

### Gene-Act-Network analysis

**Figure 5:**
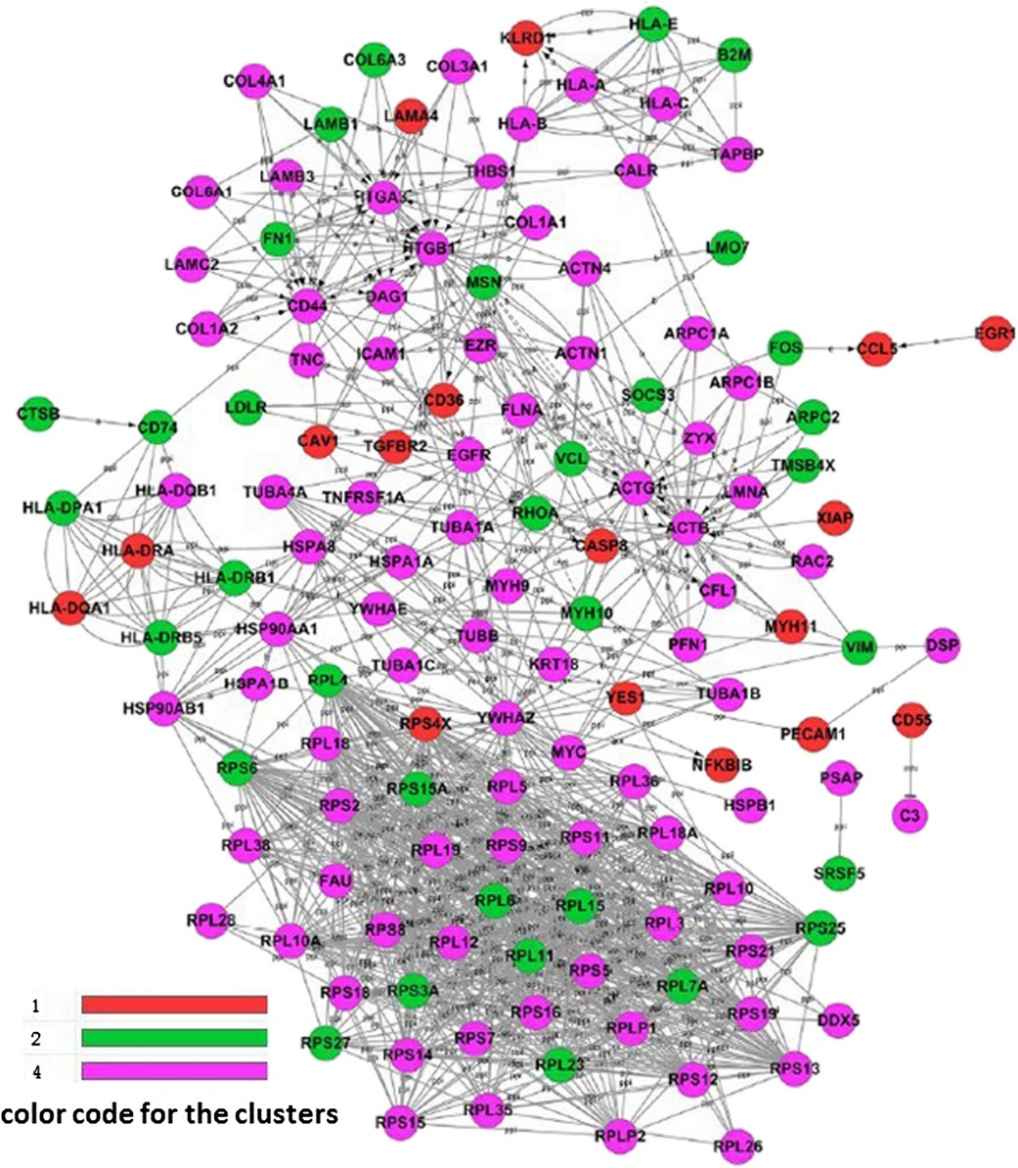
Gene-Act-Network analysis. Genes in red belong to cluster 1, genes in green belong to cluster 2, and genes in pink belong to cluster 4.

The Gene-Act-Network was established for the key genes and was suggested to be the significant genes by GO analysis. The network data for the genes in clusters 1, 2, and 4 are shown in Figure 5. *ACTN4*, as one of the genes in cluster 4, also is presented in the Gene-Act-Network.

### qRT-PCR validation for ACTN4 expression in these three types of tissues

**Figure 6:**
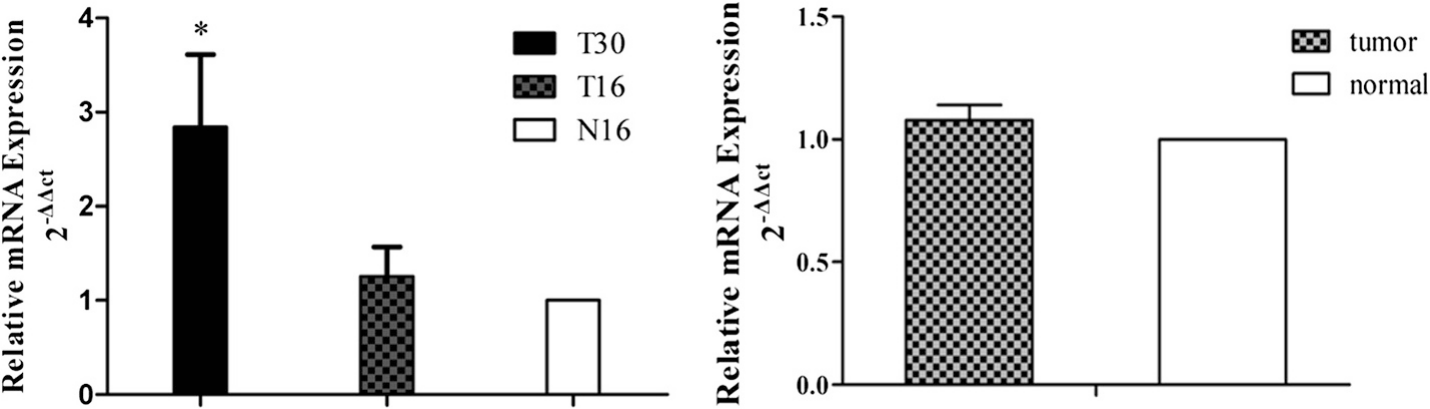
qRT-PCR validation of *ACTN4* expression in primary lung cancer, benign, and metastatic brain tissues. The expression level of *ACTN4* is higher in metastatic brain tissues than in primary lung cancer and benign tissues.

Given the significant alteration of *ACTN4* expression as indicated by the above data, the expression levels of *ACTN4* in the three types of tissues were validated using qRT-PCR. The data confirmed the RNA-Seq results and showed the significantly increased expression of *ACTN4* in the brain tumor tissues but comparable levels between primary lung cancer and the adjacent benign lung tissues of 10 cases of independent samples (Figure 6).

## Discussion

The expression profiles of primary lung cancer, adjacent benign lung tissues, and brain metastatic tumor tissues from a single patient were explored using the RNA-Seq technique. A series of bioinformatic analyses revealed gene functions and signaling pathways essential for lung cancer development and brain metastasis. Among these significant genes and pathways, *ACTN4*, encoding a nonmuscle actin cytoskeleton protein, and the pathway involved in regulation of actin cytoskeleton appeared to play a pivotal role in the process of lung cancer metastasis to the brain.

Overall, the quality of the RNA-Seq data and read mapping in our current study met the requirements for the bioinformatic analyses. However, the total reads and the mapping rate for the adjacent benign tissue were not as good as those of the primary lung cancer and the metastatic brain tumor tissues, probably resulting from the limited amount of the benign tissue and the total RNA available for the RNA-Seq approach. Nevertheless, appropriate bioinformatic analyses were performed on the RNA-Seq data from this study.

Differential gene expression analysis based on the RPKM values with upper-quartile normalization revealed that many genes were differentially expressed among these three types of tissues. The RPKM values refer to the reads per kb per million reads, according to a previous study [25]. These differentially expressed genes were classified into eight clusters according to their changed expression patterns in these three types of tissues. The genes in cluster 4 are likely candidate genes that are indispensable for lung cancer metastasis to the brain because these genes presented a significantly increased expression in the metastatic brain tumors but not in the primary lung cancer tissue or the benign tissue. The genes in cluster 4 involve a variety of cellular functions, including cytoskeleton-dependent intracellular transport, membrane-to-membrane docking, actin filament bundle assembly, cell-cell adhesion, and actin cytoskeleton organization. The pathway analysis showed similar results for the cluster 4 genes. These genes are mostly involved in the signaling pathways associated with extracellular molecular interaction, cellular adhesion, adherens junction, and cytoskeleton organization. Interestingly, *ACTN4*, encoding the alpha-actinin-4 protein, is among cluster 4 genes. Alteration of ACTN4 expression was further validated in these three types of tissues. ACTN4 has been shown to play important roles in cytoskeleton organization, cell adhesion, and cell migration. It has been suggested that ACTN4 is indispensable for mononuclear phagocyte response both in inflammation and tumor invasion processes [26]. A recent study also supported that ACTN4, particularly relying on its C-terminal tail, mediates the cytoskeleton to the adhesion site during cell migration [27]. Our results demonstrated that these functions of ACTN4 contribute to the process of lung cancer metastasis to the brain.

Of note, another alpha-actinin gene, *ACTN1*, also appeared in cluster 4 and presented a similar altered expression pattern as *ACTN4* in these three types of tissues. Indeed, *ACTN1* has been reported to be essential for cytoskeleton organization and cell motility in some types of cells [28]. Foley and Young recently have shown that ACTN4 and ACTN1 form a heterodimer in many types of cells [29]. However, it also has been shown that *ACTN1* and *ACTN4* contribute to distinct malignant properties of astrocytoma cells and that *ACTN4* may be more important for cell motility and cell adhesion in some cell lines [11].

In contrast to the functions of the genes in cluster 4, the genes in cluster 1 presented distinct functions such as regulation of reactive oxygen species, response to interlukin-6, and regulation of the activation of JAK2 kinase activity. Accordingly, the pathway analysis results were also distinct for the genes in cluster 1 and cluster 4. These results support our hypothesis that the genes in cluster 1 likely include candidate genes critical for tumorigenicity and that the genes in cluster 4 likely include candidate genes indispensable for metastasis.

The Pathway-Act-Network analysis based on the RNA-Seq data from the three types of tissues suggested that the pathways associated with the regulation of actin cytoskeleton are the pivotal players during lung cancer metastasis to the brain. Our data indicated that alteration of these actin cytoskeleton pathways could contribute to lung cancer metastasis to the brain through interacting with several other pathways involved in cellular processes, such as focal adhesion, adherens junction, and ECM-receptor interaction. The important function of the cytoskeleton in cancer metastasis has been widely recognized [30]. The results from a study of transendothelial migration of small cell lung cancer cells across human brain microvascular endothelial cells showed that the Rho/ROCK pathway contributes to actin cytoskeleton reorganization [31]. Consistent with this report, *ras homolog family member C* (*RHOC*) also appeared among the genes in cluster 4, and its expression was increased in the brain metastatic tumor but not in the primary lung cancer tissue. *ACTN4* is a critical gene related to actin cytoskeleton regulation and has been reported by multiple studies to play an important role in cell adhesion, cell motility, and cancer metastasis [4,5,12,15,17,18]. It also has been suggested that *ACTN4* may interact with *Rho* family members to regulate cell motility and cancer metastasis through regulating cytoskeleton organization and focal adhesion [11,27,28,30,31]. In short, the bioinformatic analysis data revealed that the pathways involved with actin cytoskeleton regulation were pivotal pathways in the Pathway-Act-Network and that the *ACTN4* gene was one of the key players in the Gene-Act-Network. Our current data are consistent with many previous studies, including a microarray and immunostaining data on *ACTN4* and its association with pathways contributing to lung cancer metastasis [8,10]. Our study provided the first RNA-Seq data to support the essential function of the *ACTN4* gene and the relevant cytoskeleton organization pathways in the brain metastasis of lung carcinoma. However, the fact that only one patient’s samples were used is a major limitation of our current study. A future study with more samples will help to confirm and support our current findings.

## Conclusions

In summary, the expression profiles of the primary lung cancer, adjacent benign lung tissue, and metastatic brain tissue from one patient were established using an RNA-Seq assay, and subsequent bioinformatic analyses demonstrated that the actinin gene *ACTN4* and the pathways involved in the regulation of cytoskeleton organization, cell motility, and focal adhesion are indispensable for the process of lung cancer metastasis to the brain. *ACTN4* contributes to the brain metastasis of lung cancer mainly through regulating actin cytoskeleton organization, cell motility, and focal adhesion.

## Consent

Written informed consent was obtained from the patient for publication of this article and any accompanying images. A copy of the written consent is available for review by the Editor of this journal.

## Additional files

Additional file 1: Table S1. RPKM values for the three types of tissues.
Additional file 2: Table S2. Differentially expressed genes in primary lung cancer vs. benign lung tissues.
Additional file 3: Table S3. Differentially expressed genes in primary lung cancer vs. metastatic brain tissues.
