# Supplementary material for: ACTN4 and the pathways associated with cell motility and adhesion contribute to the process of lung cancer metastasis to the brain
Source: BMC Cancer. 2015 Apr 12;15:277. doi: 10.1186/s12885-015-1295-9 (PMC4409712; doi:10.1186/s12885-015-1295-9)
Supplement: Additional file 2: Table S2. — Differentially expressed genes in primary lung cancer vs. benign lung tissues. [file 12885_2015_1295_MOESM2_ESM.doc]

**Table S2. Differentially expressed genes in primary lung cancer vs. benign lung tissues**

| **Gene Symbol** | **Fold Change** | **Regulation** | **P-value** | **FDR** |
| --- | --- | --- | --- | --- |
| KRT19 | 189.3180816 | up | 1.79834E-35 | 1.2848E-32 |
| KRT6A | 99.76429345 | up | 4.92228E-22 | 1.87555E-19 |
| HSPA1B | 88.89132082 | up | 1.09145E-25 | 5.1985E-23 |
| KRT7 | 84.56162243 | up | 6.05488E-56 | 6.01855E-53 |
| HSPA1A | 81.16865431 | up | 2.40863E-34 | 1.61959E-31 |
| GAPDH | 74.41688645 | up | 2.4833E-189 | 7.0966E-186 |
| TNS4 | 63.48855997 | up | 1.67514E-15 | 4.20846E-13 |
| LAMB3 | 63.43487166 | up | 1.69084E-14 | 4.02667E-12 |
| SPINT2 | 58.39473974 | up | 8.32184E-14 | 1.76161E-11 |
| RAB25 | 47.67022397 | up | 3.38685E-14 | 7.5912E-12 |
| DHCR24 | 47.50857582 | up | 2.00721E-14 | 4.63523E-12 |
| FAU | 45.1666237 | up | 2.49738E-11 | 4.16752E-09 |
| PLXNB2 | 42.75441996 | up | 7.5937E-09 | 9.33372E-07 |
| KRT8 | 41.9108098 | up | 1.88484E-13 | 3.88208E-11 |
| KRT18 | 39.3458761 | up | 4.00033E-17 | 1.12908E-14 |
| EGFR | 33.95391305 | up | 3.44208E-21 | 1.22957E-18 |
| HIST1H1E | 33.75100062 | up | 4.27427E-14 | 9.396E-12 |
| C17orf28 | 33.72218772 | up | 3.95785E-08 | 4.22824E-06 |
| ACTN1 | 33.45275103 | up | 4.49138E-20 | 1.53257E-17 |
| ALDOA | 32.45284295 | up | 7.32292E-12 | 1.28782E-09 |
| HIST1H3B | 32.16555684 | up | 3.81111E-07 | 3.3383E-05 |
| ITGA3 | 32.04202869 | up | 1.68853E-07 | 1.55658E-05 |
| ENO1 | 30.58300468 | up | 8.36563E-11 | 1.30997E-08 |
| ECH1 | 30.18682184 | up | 6.64535E-30 | 3.70551E-27 |
| SERINC2 | 29.83992314 | up | 4.46871E-08 | 4.66501E-06 |
| MT1E | 29.41196674 | up | 7.73637E-13 | 1.5117E-10 |
| SLC25A39 | 29.32900958 | up | 1.15586E-08 | 1.35514E-06 |
| TACC3 | 27.88801629 | up | 1.12519E-06 | 8.69059E-05 |
| LGALS3BP | 27.20067736 | up | 2.18794E-11 | 3.70523E-09 |
