# Supplementary material for: ACTN4 and the pathways associated with cell motility and adhesion contribute to the process of lung cancer metastasis to the brain
Source: BMC Cancer. 2015 Apr 12;15:277. doi: 10.1186/s12885-015-1295-9 (PMC4409712; doi:10.1186/s12885-015-1295-9)
Supplement: Additional file 3: Table S3. — Differentially expressed genes in primary lung cancer vs. metastatic brain tissues. [file 12885_2015_1295_MOESM3_ESM.doc]

**Table S3. Differentially expressed genes in primary lung cancer vs. metastatic brain tissues**

| **Gene Symbol** | **Fold Change** | **Regulation** | **P-value** | **FDR** |
| --- | --- | --- | --- | --- |
| IGKV4-1 | 30.24485644 | up | 5.92302E-18 | 1.75007E-15 |
| RN18S2P | 11.13810067 | up | 3.00695E-15 | 7.76236E-13 |
| IGKV3-20 | 8.284477435 | up | 2.54791E-07 | 2.6365E-05 |
| IGKV3-15 | 8.268349968 | up | 0.00060377 | 0.020679966 |
| RN7SL1 | 7.836716985 | up | 1.60119E-08 | 2.08871E-06 |
| RN7SL2 | 6.59566844 | up | 6.17168E-08 | 7.17319E-06 |
| IGKC | 6.144891623 | up | 1.0315E-26 | 4.2161E-24 |
| RNR1 | 3.899404756 | up | 0 | 0 |
| RNR2 | 2.907755779 | up | 0 | 0 |
| IL34 | 0.486688317 | down | 0.000226379 | 0.009330606 |
| LOC100506792 | 0.474819914 | down | 0.00098363 | 0.029854638 |
| DTX2 | 0.472968522 | down | 0.000955051 | 0.029277103 |
| ND6 | 0.470118693 | down | 4.6911E-236 | 9.5871E-233 |
| PCBD2 | 0.469176644 | down | 1.48113E-07 | 1.60014E-05 |
| ST13P20 | 0.466357079 | down | 0.000317581 | 0.012602532 |
| CYB5R3 | 0.457164398 | down | 0.000138095 | 0.006179989 |
| MTND2P28 | 0.449014448 | down | 4.00132E-94 | 4.46038E-91 |
| LINC-ROR | 0.436061446 | down | 8.0678E-10 | 1.27648E-07 |
| TRIM26 | 0.434932788 | down | 0.000255843 | 0.010405149 |
| LOC100507511 | 0.431650088 | down | 0.000661376 | 0.022188203 |
| PTMA | 0.431617328 | down | 9.80386E-06 | 0.000679181 |
| HIPK2 | 0.431289614 | down | 1.53976E-08 | 2.04114E-06 |
| ACTB | 0.429960144 | down | 7.50581E-25 | 2.87613E-22 |
| RPL41 | 0.426868752 | down | 0.00011744 | 0.005403585 |
| SHROOM1 | 0.424273765 | down | 0.001347323 | 0.037167321 |
| ND2 | 0.424063915 | down | 0 | 0 |
| LOC100506334 | 0.422609595 | down | 2.2479E-07 | 2.36599E-05 |
| ND3 | 0.419379541 | down | 1.1316E-36 | 6.03291E-34 |
| IDO1 | 0.419351474 | down | 7.70447E-05 | 0.003809361 |
